# The Contribution of Sociocultural Factors in Shaping Self-Reported Sickness Behavior

**DOI:** 10.3389/fnbeh.2020.00004

**Published:** 2020-01-24

**Authors:** Eric C. Shattuck, Jessica K. Perrotte, Colton L. Daniels, Xiaohe Xu, Thankam S. Sunil

**Affiliations:** ^1^Institute for Health Disparities Research, College of Liberal and Fine Arts, The University of Texas at San Antonio, San Antonio, TX, United States; ^2^Department of Psychology, Texas State University, San Marcos, TX, United States; ^3^Department of Sociology, College of Liberal and Fine Arts, The University of Texas at San Antonio, San Antonio, TX, United States; ^4^School of Public Administration, Sichuan University, Chengdu, China

**Keywords:** sickness behavior, stoicism, gender role, machismo, familism, individualism, collectivism, inflammation

## Abstract

Sickness behavior is an evolutionarily conserved phenomenon found across a diverse range of animals involving a change in motivational priorities to theoretically maximize energetic investment in immune function and recovery. Typical components of sickness behavior include reduced sociability and activity, changes in diet, and depressed affect. Importantly, however, sickness behavior appears to be subject to other demands of life history in animal models, including reproduction and offspring survival. Thus, “feeling sick” is often context dependent with possible effects on morbidity and mortality. While humans may not always face the same life history trade-offs, sociocultural norms and values may similarly shape sickness behavior by establishing internalized parameters for “socially appropriate sickness.” We explore the role of these factors in shaping sickness behavior by surveying a national U.S. sample (*n* = 1,259). Self-reported and recalled sickness behavior was measured using the SicknessQ instrument, which has previously been validated against experimentally induced sickness behavior. After post-stratification weighting and correction for Type I error, generalized linear models showed that sickness behavior is significantly affected by various factors across sex and racial/ethnic groupings. Income below the national mean (*b* = 1.85, adj. *p* = 0.025), stoic endurance of pain and discomfort (*b* = 1.61, adj. *p* < 0.001), and depressive symptomology (*b* = 0.53, adj. *p* < 0.001) were each associated with greater sickness behavior scores. Familism (*b* = 1.59, adj. *p* = 0.008) was positively associated with sickness behavior in men, but not women. Endurance of pain and discomfort was associated with greater sickness behavior in Whites only (*b* = 1.94, adj. *p* = 0.002), while familism approached significance in African Americans only (*b* = 1.86, adj. *p* = 0.057). These findings may reflect different social contexts of sickness across demographic groups, which may in turn have important implications for pathogen transmission and recovery times, potentially contributing to health disparities.

## Introduction

During sickness, regular behavioral patterns are temporarily suspended in favor of a general reduction in activity, a phenomenon known as sickness behavior. This type of behavioral change is an organized shift in motivational priorities on the part of an infected host, resulting in a suite of changes in affect and behavior (Aubert, 1999). In addition to reduced activity, other changes include social withdrawal, decreased libido, reduced food intake, and cognitive disruptions, among others. Sickness behavior has been observed under experimental and natural conditions in a variety of animal species, from honeybees (*Apis mellifera*; Kazlauskas et al., 2016) to humans (e.g., Eisenberger et al., 2010; Smith, 2012; de Goeij et al., 2013; Lasselin et al., 2019). It is thought that these changes support energetically expensive immune responses by reducing overall energy expenditure, thereby leading to shorter or less severe infections (Hart, 1988). Mechanistically, sickness behavior is driven by several pro-inflammatory cytokines, namely interleukins-1β (IL-1β) and IL-6 and tumor necrosis factor-α (TNF-α) (McCusker and Kelley, 2013) with a likely role for prostaglandins PGE2 and PGD2 (Saper et al., 2012). These cytokines are produced as part of the acute phase response (APR), a cornerstone of the innate arm of the immune system and the earliest response to injury or infection (Baumann and Gauldie, 1994). The APR results in several physiological changes critical to an effective immune response, including fever and leukocytosis. Sickness behavior is therefore deeply intertwined with immune responses.

There is a body of research suggesting sickness behavior is also responsive to social and environmental contexts. Some animal studies, for instance, suggest that sickness behavior may not be enacted when threats to survival or opportunities for reproduction are present. Captive rhesus macaques (*Macaca mulatta*) injected with recombinant human IL-1α showed none of the expected changes in alertness or somnolence associated with sickness behavior when faced with a threatening behavior (i.e., a researcher maintaining eye contact with them; Friedman et al., 1996). Aubert et al. (1997) demonstrated that threats to offspring survival led to a resumption of normal maternal behaviors in mouse dams during experimental sickness behavior. Similar experiments have shown that sickness behavior ceased when male zebra finches (*Taeniopygia guttata*) were presented with a novel female (Lopes et al., 2013). These and similar findings highlight the contextual nature of sickness behavior, which appears to be enacted only when the benefits of acting sick outweigh any potential opportunity costs (Lopes, 2014).

The complex social and cultural lives characteristic of humans may similarly influence sickness behavior expression and create new trade-offs, with possible consequences for health and pathogen transmission. For instance, several studies have found that doctors and other healthcare practitioners often counterintuitively work while symptomatic. In one such study, approximately 91–93% of healthcare practitioners attended work while symptomatic for influenza-like illnesses (Mossad et al., 2017). Another study found that 83% of surveyed practitioners reported working with diarrhea, fever, and other symptoms, although knowing this posed a risk to patients and co-workers (Szymczak et al., 2015). Because sickness behavior is so intimately linked with inflammation and the acute-phase response, it is reasonable to assume that these healthcare practitioners felt the influence of sickness behavior on their motivation to work. However, they continued with normal behaviors instead of heeding this biological cue. Common reasons given for working while sick include structural concerns such as not wanting to burden colleagues with additional work, staffing concerns, and unsupportive supervisors, as well as strong cultural norms in their hospital to continue working unless one is extremely ill (Szymczak et al., 2015). Certainly, continued presence at work while sick or symptomatic (i.e., presenteeism) is not limited to healthcare practitioners (Aronsson and Gustafsson, 2005).

Beyond workplace-specific cultures and structural factors, enacting sickness behavior could be shaped by individual level factors that influence how sickness symptoms are defined, given significance, and acted upon. Research in psychology, sociology, and similar fields has found that a diverse array of internalized attitudes and beliefs can affect symptom interpretation, reporting, and healthcare seeking. For instance, endorsing both lower masculinity and higher femininity have been associated with more reported physical, mental, and psychophysical symptoms in men and women (Annandale and Hunt, 1990). Other research indicates that individuals with more stereotypically traditional feminine characteristics across identity and behavioral domains (e.g., endorsing more feminine identity, spending more time doing housework, earning less income) were more likely to experience recurrent acute coronary syndrome (ACS), independent of biological sex (Pelletier et al., 2016). The authors speculate that this is due to stressful or burdensome gender role expectations.

Other gender role prescriptions may inform whether a person actually engages – or the degree to which they engage – in sickness behavior when ill. Stoical beliefs have been linked with underreporting of pain (Yong, 2006) and slow or delayed healthcare seeking (MacLean et al., 2017). Notably, although stoicism is often gendered in the literature and considered a key component of masculinity, MacLean et al. (2017) found that both men and women emphasized their stoicism in the face of physical symptoms. Machismo appears to operate similarly to stoicism. This set of stereotypical beliefs about masculinity influences has been shown to affect beliefs about one’s health status in Mexican American men, in part by setting normative expectations (e.g., caring for one’s family); “illness” and impairment occur when these expectations cannot be met, according to qualitative interviews (Sobralske, 2006). A similar concept, hegemonic masculinity, predicted lower odds of engaging in preventative healthcare, including physical exams and prostate exams in a predominantly White sample (Springer and Mouzon, 2011).

In addition, beliefs about the responsibilities one has for themselves or others might shape sickness and healthcare seeking behaviors. As an example, individuals who believe that their health is reliant on God or who may view illness as a test from God might be less likely to seek treatment or to interrupt their normal activities due to illness (e.g., Gonzalez-Swafford and Gutierrez, 1983). Views about self-control over illness and willingness to reach out to others for help when sick can also vary based on individualistic and/or collectivistic beliefs (Sharp and Koopman, 2013). An emphasis on individual responsibility places the burden of healing on the sick individual, while individuals who are more embedded in social groups, whether friends or relatives, may be more willing to discuss their health and seek help from others when sick. Additionally, there are psychological benefits associated with social support and positive integration, including family, that can help to buffer the negative effects of stress on physical health. For instance, familism (i.e., the degree to which one values close family ties) has been associated with increased subjective health and decreased physical symptoms in multiple ethnicities (Corona et al., 2017) as well as greater sensitivity to the anti-inflammatory effects of IL-10 and cortisol in *ex vivo* immune cell stimulation experiments in African American and Latino, but not White, youth (Chiang et al., 2019). This last study is particularly relevant, given sickness behavior’s inflammatory basis.

Coping style has also been linked with sickness symptoms. For instance, there is evidence that maladaptive coping strategies (e.g., venting) may be correlated with experiencing more HIV symptoms (Ashton et al., 2005). Disengaged coping (e.g., denial, avoidance, and wishful thinking) being associated with greater numbers of somatic complaints, in addition to higher levels of anxiety and depression, in adolescent with recurring pain (Compas et al., 2006). Furthermore, a passive coping style was associated with worse functional outcomes in chronic illnesses (Scharloo et al., 1998). The link between coping style and sickness behavior is understudied, although it is possible that a more active coping style may relate to sickness behavior by encouraging rapid treatment of symptoms.

Whether each of these aforementioned factors affect how individuals interpret and act upon sickness behavior is not yet known, though recent results indicate that similar psychological factors contribute to symptom severity and delayed recovery during viral infection (Cvejic et al., 2019). To address this question, we conducted a nationwide survey in the United States, guided by the hypotheses that these norms and beliefs would predict variability in sickness behavior and would operate differently across sexes and ethnicities based on differences in shared norms and beliefs between these groups.

## Materials and Methods

The survey, conducted in November 2018, includes 1,259 participants. Inclusion criteria were: having been sick during the past year; being between the ages of 18 and 55 years; and being of non-Hispanic White, African American, or Hispanic descent. This study was approved by the IRB at The University of Texas at San Antonio (IRB #19-020E). Qualtrics recruited and screened participants and distributed the survey. A total of 2,815 individuals were invited to take part in the survey. Of those who agreed to participate, 519 individuals were either screened out or did not complete the survey. The final sample size was 1,259, for a response rate of 45%. Unweighted participant ethnicity and sex counts were: 429 Whites, 421 African Americans, 409 Hispanics, with 629 men and 630 women. Mean age was 36 years. The median income category was $40,000–$49,999 and median education was “Some College.” U.S. census data were used to weight the survey data by race/ethnicity, sex, and income (as poststratification weights) to reflect the larger American population. Income categories (range <$10,000 to >$150,000) were recoded as either above or below the U.S. median household income ($60,000).

To assess sickness behavior as a dependent variable, we used the SicknessQ, a 10-item measure of perceived sickness behavior that has been validated under experimental conditions (Andreasson et al., 2016). Briefly, SicknessQ scores increased from baseline following endotoxin administration and showed no significant difference relative to baseline several hours later (ibid). Sample items include, “I want to keep still,” “I feel tired,” “I wish to be alone,” and “My body feels sore.” Participants were prompted to think about recent times that they had been sick with illnesses like influenza or the common cold and to complete the SicknessQ based on how they felt. The mean unweighted SicknessQ score was 15.78 (range 0–30, SD 8.49) with a Cronbach’s alpha of 0.91. Participants were also asked to rate their current feelings of sickness from “not sick” to “severely” using a Likert-type scale (range 0–7, mean 2.07, SD 2.27) in order to control for any possible confounding effect.

Norms and beliefs of interest were gender role identity (Traditional Masculinity-Femininity Scale; Kachel et al., 2016), traditional machismo (The Traditional Machismo and Caballerismo Scale; Arciniega et al., 2008), familism (Gaines et al., 1997), the God Locus of Health Control Scale (Wallston et al., 1999), individualism and collectivism (the 14-item Individualism/Collectivism Scale; Sivadas et al., 2008), the John Henryism Active Coping Scale (James, 1994), and dimensions of stoicism (Pathak–Wieten Stoicism Ideology Scale; Pathak et al., 2017), including endurance of pain and discomfort, emotional taciturnity, and indifference to death. For gender roles, participants rated the degree to which they viewed their interests, selves, behavior, and other aspects as masculine or feminine (e.g., “Traditionally, my outer appearance would be considered as…”), with higher scores indicating greater femininity. Higher scores on the traditional machismo scale indicate a stronger adherence to this set of beliefs, including that a man should not cry in front of his children and that the father is the central figure in the family. Higher scores on the familism (e.g., “I cherish the time that I spend with my relatives.”), individualism (e.g., ‘I enjoy being unique and different from others in many ways.”), and collectivism (e.g., “I usually sacrifice my self-interest for the benefit of my group.”) scales similarly indicate a greater importance each of these set of beliefs. Higher scores on the John Henryism scale indicate a greater willingness to put forth sustained cognitive and emotional effort to confront psychosocial stressors (e.g., “When things don’t go the way I want them to, that just makes me work even harder”). Following Pathak et al. (2017) methods, the stoicism measures were centered, such that positive values indicate greater stoicism and negative values represent less. Cronbach’s alpha values for all scales showed good reliability in this sample (i.e., α range 0.72–0.97), with the exception of the stoic serenity (i.e., refraining from experiencing strong emotions, α = 0.46) and stoic death (i.e., the belief that death is not to be feared or avoided; α = 0.34) indifference domains of the Pathak–Wieten Stoicism Ideology Scale (Pathak et al., 2017) which were removed from further analyses. The vertical and horizontal dimensions of individualism and collectivism were collapsed into a single dimension by using the average scores of the vertical and horizontal subscales. The 10-item Center for Epidemiologic Studies Depression Scale (CESD-10; Andersen et al., 1994) scores were included to control for negative affect’s influence on retrospective symptom appraisal (Howren and Suls, 2011). Descriptive statistics for each of these scales are shown in Table 1.

**TABLE 1:** Means, standard deviations, and ranges for Social Norms Scales.

Scale	Mean	*SD*	Range
Traditional masculinity–femininity	4.27	2.02	1–7
Machismo	3.4	1.53	1–7
Familism	3.85	0.94	1–5
God health locus of control	19.98	9.42	6–36
Individualism	6.03	1.70	1–9
Collectivism	5.83	1.70	1–9
John Henryism	46	8.84	12–60
Stoic endurance of pain/illness	0.4	1.07	−2 to 2
Stoic taciturnity	0.44	1.06	−2 to 2
CESD	11.27	6.32	0–30

The effects of these social norms and beliefs on sickness behavior were estimated using generalized linear models, adjusted for age, sex, current feelings of sickness, marital status, and ethnicity. Models were constructed in R v. 3.5.1 (R Core Team, 2018) using the *svyglm* command of the *survey* (Lumley, 2004) package. Additionally, generalized linear models including the relevant interaction terms were also used to compare effects between men and women and ethnic categories on the *a priori* assumption that meaningful differences in sickness behavior reporting might be found based on these groupings. All *p*-values were corrected for multiple comparisons using the Benjamini–Hochberg method using the *p.adjust* function in R.

## Results

### Complete Sample

Coefficients and 95% confidence intervals for the full survey sample are presented in Table 2. Prior to correction, we found that SicknessQ scores were lower among African Americans, relative to Whites (*b* = −1.33, *p* < 0.05). Additionally, income below the national median, stronger current feelings of sickness, higher endorsement of active coping strategies (i.e., John Henryism), greater endurance of pain and discomfort, and greater current CESD scores were all associated with higher recalled SicknessQ scores (Table 2). After correction, only income (adj. *p* = 0.025), current feelings of sickness (adj. *p* = 0.008), endurance of pain and discomfort (adj. *p* < 0.01), and CESD score (adj. *p* < 0.001) remained as significant predictors.

**TABLE 2:** Full model results, coefficients, and 95% confidence intervals.

Dependent variable	SicknessQ score
Age	−0.049(−0.103,0.005)
Below median income	1.852***(0.548,3.156)
African American (ref. white)	−1.332**(−2.618,−0.045)
Hispanic (ref. white)	−0.448(−1.728,0.832)
Female (ref. male)	1.227(−0.221,2.675)
Currently married	0.081(−1.170,1.333)
Current feelings of sickness	0.427***(0.165,0.688)
Gender role	0.298(−0.067,0.662)
Machismo	−0.195(−0.676,0.286)
Familism	0.687(−0.010,1.383)
John Henryism	0.086**(0.011,0.162)
God health locus of control	−0.015(−0.085,0.056)
Individualism	0.291(−0.118,0.700)
Collectivism	−0.158(−0.627,0.310)
Endurance of pain/discomfort	1.614***(0.914,2.314)
Taciturnity	−0.059(−0.746,0.627)
CESD	0.526***(0.437,0.616)
Constant	0.957(−3.359,5.273)
Adjusted *R*^2^	0.353
Log likelihood	−4,421.961
Akaike Information Criterion	8,879.922

**p < 0.05; ***p < 0.01.

### Differences Between Sexes

Table 3 shows the results of the same model including an interaction term for sex, with male as the reference category. Greater endurance of pain or discomfort remained positively associated with sickness behavior in both sexes, though the relationship was weaker in women (*b* = 1.261 vs. *b* = 3.17 in men, *p* < 0.001 in both). In men only, familism was positively associated with greater SicknessQ scores (*b* = 1.59, *p* < 0.001). After correction, familism (adj. *p* = 0.008), endurance of pain and discomfort (adj. *p* < 0.001), and CESD scores (adj. *p* < 0.001) remained significant predictors in men, whereas endurance of pain and discomfort was the only significant predictor in women (adj. *p* < 0.004).

**TABLE 3:** Model results including interaction for sex (ref. male), coefficients, and 95% confidence intervals.

Dependent variable	SicknessQ score
Age	−0.046(−0.127,0.035)
Below median income	2.052**(0.417,3.687)
African American (ref. white)	−0.239(−2.160,1.682)
Hispanic (ref. white)	0.758(−1.109,2.625)
Currently married	0.300(−1.435,2.034)
Current feelings of sickness	0.314(−0.026,0.653)
Gender role	0.526**(0.042,1.010)
Machismo	−0.014(−0.690,0.663)
Familism	1.597***(0.653,2.540)
John Henryism	0.055(−0.052,0.161)
God health locus of control	−0.057(−0.161,0.048)
Individualism	0.080(−0.566,0.726)
Collectivism	−0.081(−0.765,0.602)
Endurance of pain/discomfort	3.171***(2.282,4.059)
Taciturnity	0.055(−0.867,0.976)
CESD	0.374***(0.264,0.485)
Sex	5.785(−2.270,13.839)
Age * sex	−0.004(−0.109,0.101)
Below median income * sex	−0.420(−2.774,1.935)
African American (ref. white) * sex	−0.985(−3.525,1.554)
Hispanic (ref. white) * sex	−1.568(−4.057,0.922)
Currently married * sex	−0.195(−2.463,2.073)
Current feelings of sickness * sex	0.105(−0.384,0.593)
Gender role * sex	−0.390(−1.148,0.367)
Machismo * sex	−0.519(−1.438,0.401)
Familism * sex	−1.272**(−2.540,−0.004)
John Henryism * sex	0.040(−0.104,0.185)
God health locus of control * sex	0.046(−0.088,0.180)
Individualism * sex	0.207(−0.602,1.017)
Collectivism * sex	−0.101(−0.984,0.783)
Endurance of pain/discomfort * sex	−2.325***(−3.586,−1.064)
Taciturnity * sex	−0.140(−1.418,1.138)
CESD * sex	0.199**(0.045,0.353)
Constant	−0.752(−6.229,4.724)
Adjusted *R*^2^	0.376
Log likelihood	−4,391.599
Akaike Information Criterion	8,851.199

**p < 0.05; ***p < 0.01.

### Differences Between Ethnic Categories

Model results are presented in Table 4. Among non-Hispanic Whites, CESD scores and endurance of pain or discomfort were positively associated with sickness behavior. Among Hispanics, machismo was a significant negative predictor (*b* = −1.201, *p* < 0.001) of SicknessQ scores, relative to whites. Among African Americans, familism (*b* = 2.068, *P* < 0.001) was associated with stronger sickness behavior. After correction, CESD and endurance of pain or discomfort remained significant in non-Hispanic Whites (adj. *p* < 0.001 and = 0.0025, respectively). In the other ethnic groups, only familism in African Americans approached significance after correction (adj. *p* = 0.057).

**TABLE 4:** Model results including interaction for ethnicity, coefficients, and 95% confidence intervals.

Dependent variable	SicknessQ score
Age	−0.070(−0.145,0.005)
Below median income	1.827(−0.051,3.706)
Female (ref. male)	1.381(−0.653,3.416)
Currently married	−0.248(−2.010,1.514)
Current feelings of sickness	0.346(−0.016,0.708)
Gender role	0.361(−0.150,0.871)
Machismo	0.116(−0.540,0.772)
Familism	0.204(−0.629,1.037)
John Henryism	0.026(−0.075,0.128)
God health locus of control	0.011(−0.084,0.106)
Individualism	0.162(−0.356,0.680)
Collectivism	−0.085(−0.736,0.565)
Endurance of pain/discomfort	1.937***(0.936,2.939)
Taciturnity	−0.312(−1.241,0.617)
CESD	0.374***(0.264,0.485)
African American (ref. white)	−15.728***(−23.765,−7.692)
Hispanic (ref. white)	−5.225(−13.692,3.242)
Age * African American	0.074(−0.026,0.174)
Age * Hispanic	0.062(−0.044,0.168)
Below median income * African American	−0.288(−3.144,2.567)
Below median income * Hispanic	−1.089(−4.043,1.865)
Female (ref. male) * African American	−0.318(−3.201,2.564)
Female (ref. male) * Hispanic	−0.567(−3.548,2.415)
Currently married * African American	−0.636(−3.309,2.036)
Currently married * Hispanic	1.156(−1.295,3.607)
Current feelings of sickness * African American	0.343(−0.170,0.856)
Current feelings of sickness * Hispanic	0.022(−0.506,0.551)
Gender role * African American	0.119(−0.595,0.832)
Gender role * Hispanic	−0.170(−0.985,0.645)
Machismo * African American	−0.255(−1.193,0.684)
Machismo * Hispanic	−1.317***(−2.306,−0.328)
Familism * African American	1.864***(0.571,3.157)
Familism * Hispanic	0.532(−0.702,1.765)
John Henryism * African American	0.139(−0.0001,0.279)
John Henryism * Hispanic	0.159(−0.002,0.321)
God health locus of control * African American	−0.064(−0.199,0.070)
God health locus of control * Hispanic	−0.065(−0.211,0.080)
Individualism * African American	0.494(−0.343,1.331)
Individualism * Hispanic	0.316(−0.554,1.185)
Collectivism * African American	−0.431(−1.366,0.504)
Collectivism * Hispanic	−0.230(−1.193,0.733)
Endurance of pain/discomfort * African American	−1.318(−2.702,0.066)
Endurance of pain/discomfort * Hispanic	−0.729(−2.272,0.814)
Taciturnity * African American	0.609(−0.717,1.934)
Taciturnity * Hispanic	1.019(−0.547,2.585)
CESD * African American	−0.039(−0.219,0.142)
CESD * Hispanic	−0.069(−0.235,0.098)
Constant	5.094(−1.082,11.270)
Adjusted *R*^2^	0.376
Log likelihood	−4,393.280
Akaike Information Criterion	8,882.560

**p < 0.05; ***p < 0.01.

## Discussion

The results from this large, nationwide survey suggest that self-reported, recalled sickness behavior can be shaped by numerous factors, such as demographics, social norms, and affect. Furthermore, there is evidence that these factors operated differently by sex and ethnicity. Income below the national median, stoicism, familism, and depression were each associated with stronger sickness behavior in different sex and ethnic groups.

Income below the U.S. national median was associated with increased self-reported sickness behavior in the full sample. Lower SES has been consistently linked with increased risk of morbidity and mortality (Adler et al., 1994), as well as the number and severity of self-reported physical symptoms (Zilioli et al., 2017). Similar to our results, Cvejic et al. (2019) found that low SES was associated with greater fatigue, pain, and mood disturbance in individuals suffering from acute viral illnesses. In terms of sickness behavior, lower SES individuals in our sample may not have sought to alleviate their symptoms as early as their counterparts and allowed their most recent bout of sickness to become more severe. Following Zilioli et al. (2017), higher SicknessQ scores may also be related to accumulated stress and a lack of perceived control among low SES individuals. While our study did not measure perceived control, active coping (i.e., John Henryism), which might reflect greater perceived control over stressors, is associated with more severe sickness behavior, albeit only prior to correction. In the original formulation of the John Henryism hypothesis (James, 1994), a more active coping style was thought to lead to worse health outcomes as the result of increased physical and mental effort spent to counteract stressors. Under experimental conditions, stress has been shown to worsen the physical and psychological effects of inflammation (Brydon et al., 2009). Thus, greater stress experienced by low SES individuals and those higher in active coping could lead to worse physical symptoms.

Depressive symptoms were positively associated with symptom severity in our study, in accordance with research showing that depressive disorders are linked with somatic symptoms (e.g., Bekhuis et al., 2015) and, through increased memory of negative events, with greater retrospective and concurrent symptom reporting (Neitzert et al., 1997; Howren et al., 2009). Conversely, positive emotions have been associated with decreased illness rates after experimental infection with either rhinovirus or influenza (Cohen et al., 2006) as well as fewer physical symptoms of illness, such as congestion and sore throat (Doyle et al., 2006). Thus, it is possible that individuals endorsing more depressive symptoms in our sample may have been more vulnerable to minor infections and so had a more recent (and therefore more salient) sickness, have better recall of past symptoms, or have more severe symptoms in general. Note that these possible explanations are not necessarily mutually exclusive.

In the full sample, in men and women, and in non-Hispanic Whites, a stoic endurance of pain or illness was associated with stronger self-reported sickness behavior, contrary to many reports of reduced symptom reporting and/or pain among highly stoic individuals (e.g., Yong, 2006; Murray et al., 2008). One possible explanation for our findings is that individuals who perceive themselves as particularly stoic in their response to pain and discomfort overestimate past sickness behavior symptoms due to recall bias. Alternatively, this kind of stoicism may be highly valued by some groups in the United States, leading participants to over-report their stoicism score. There is also considerable research (albeit within the framework of masculinity) on the role of stoicism in significant or chronic illnesses, such as depression (see, e.g., Seidler et al., 2016 for a review) and chronic pain (Yong, 2006). However, our survey prompted participants to discuss their experience(s) with the common cold, flu, and other minor illnesses. There is likely less stigma in reporting influenza-like symptoms than depression or other major illnesses, so our participants may have been more forthcoming. Increased symptom severity may also reflect reduced healthcare seeking among stoic individuals (again largely framed in terms of masculinity; O’Brien et al., 2005; Jeffries and Grogan, 2012). Stoic individuals in our sample may simply have not used over-the-counter medications to control symptoms or sought medical attention until their illness was more advanced.

Familism has largely been associated with better physical and mental health, particularly in Hispanic/Latino individuals (Valdivieso-Mora et al., 2016; Corona et al., 2017), although this relationship has been found in other ethnicities (Campos et al., 2014). As with stoicism, our findings run contrary to others. That is, increased self-reported sickness behavior was positively associated with familism in men and African Americans, although this latter relationship falls short of conventional statistical significance after correction. Our results may also be due to differences between sickness behavior and other health outcomes, such as psychological health, measured in previous research. Our findings may also indicate that a social “safety net” is an important factor for expressing sickness behavior. Some researchers have hypothesized that some physical symptoms may serve as a signal to family and group members to elicit care (Fabrega, 1997; Steinkopf, 2015; Tiokhin, 2016). It may be that, in individuals who maintain a closer link (either geographically or emotionally) with their family, stronger short-term sickness behavior symptoms may result in quicker care or emotional support, thereby leading to faster recovery. Additional research to explicitly test this hypothesis is necessary, however. This form of social support may be particularly important for men, who are often socialized to be stoic in the face of sickness, as noted above. Intriguingly, familism was associated with greater SicknessQ scores only among African Americans in our study. In a large survey of young adults, Schwartz et al. (2010) found that African Americans scored highest on measures of collectivist values, including familism. They note that, given a long history of discrimination, African Americans have largely maintained a separate cultural identity and that collectivist values may therefore function more strongly than in other ethnicities, though they may endorse similar values (Schwartz et al., 2010). Thus, collectivist values, including familism, may have a stronger bearing on behaviors in multiple domains, including health and sickness, in African Americans relative to other groups.

These results should be interpreted cautiously in light of several study limitations. For one, the SicknessQ was developed and validated to measure sickness behavior in response to endotoxin among adults in Sweden (Andreasson et al., 2016). To the best of our knowledge, our study is the first to apply the SicknessQ to recalled sickness behavior, and the first use the measure to assess sickness behavior among ethnic and racially diverse adults in the United States. The SicknessQ is the most judicious choice to address sickness behavior currently available, as it is the only instrument specifically designed to measure sickness behavior, and it has been validated against the biological basis of the phenomenon (i.e., inflammation). Another limitation is that we do not have information about medication use in this sample. Medication, particularly anti-inflammatories, could lead to reduced SicknessQ scores independent of any effect of sociocultural factors. Finally, our prompt asked participants to think about their experiences with colds, the flu, and other illnesses. It is possible that variation in the severity of illnesses could lead to heterogeneity in SicknessQ scores. Notably, a variety of modalities have been used to study aspects of sickness behavior in humans, including the use of endotoxin (e.g., Lasselin et al., 2018), tetanus vaccination (Brydon et al., 2009), and natural infection with respiratory viruses (e.g., Smith et al., 1987; Smith, 2012) and Ross River virus, Epstein–Barr virus, and Q fever (Cvejic et al., 2019). While a general pattern of sickness behavior (e.g., social withdrawal, cognitive disturbances, etc.) occurs across these disparate conditions, pathogen-dependent differences in sickness behavior severity are unexplored in research.

Although these results cannot directly assess whether or not human sickness behavior at a biological level is subject to the same opportunity cost trade-offs observed in animal models, we show preliminary evidence that sickness behavior symptom reporting may be shaped by multiple beliefs and social norms across different demographic groups. In particular, sickness behavior reporting may coincide with the perceived ability to more freely enact sickness behavior, as in the case of greater familism in men and African Americans. Conversely, those who value being stoic in the face of pain and discomfort may report less sickness behavior. Further research using experimentally induced inflammation can clarify whether these findings, which rely on recall and self-report, are found during active sickness or inflammation and whether ignoring sickness behavior comes with any health costs. Ethnographic research can further explore how an individual’s beliefs and life contexts shape their behavioral responses to minor infectious diseases, providing a level of detail and nuances that surveys cannot capture.

Understanding how sociocultural factors can influence an evolved behavioral response that forms a fundamental, albeit under-appreciated, component of our overall immune response has numerous practical implications. For instance, at the individual level, it is possible that ignoring or suppressing sickness behavior results in longer or more severe infections. Additionally, if sickness behavior also serves to signal to friends and family that care and support are necessary, suppressing these visible symptoms may delay receiving help, perhaps again resulting in longer or more severe illness. At the societal level, infected individuals can transmit pathogens to healthy coworkers, classmates, friends, and family. Personality and cultural factors that deter enacting sickness behavior, such as stoicism, may be encouraging sick individuals to ignore an evolved biological signal at the risk of larger and prolonged transmission chains.

## Data Availability Statement

The raw data used for this study will be made available by the corresponding author to any qualified researcher without undue reservation.

## Ethics Statement

The studies involving human participants were reviewed and approved by the Institutional Review Board, The University of Texas at San Antonio. Written informed consent for participation was not required for this study in accordance with the national legislation and the institutional requirements.

## Author Contributions

ES, TS, and XX conceived and designed the study. ES and CD organized the database. ES, JP, CD, TS, and XX conceived the statistical approach. ES performed the statistical analysis and wrote the manuscript. All authors contributed to the manuscript revision, and read and approved the submitted version of the manuscript.

## Conflict of Interest

The authors declare that the research was conducted in the absence of any commercial or financial relationships that could be construed as a potential conflict of interest.
